# The Ability of Porcine Reproductive and Respiratory Syndrome Virus Isolates to Induce Broadly Reactive Neutralizing Antibodies Correlates With *In Vivo* Protection

**DOI:** 10.3389/fimmu.2021.691145

**Published:** 2021-07-26

**Authors:** Francisco Javier Martínez-Lobo, Francisco Díez-Fuertes, Isabel Simarro, José M. Castro, Cinta Prieto

**Affiliations:** ^1^ Animal Science Department, School of Agrifood and Forestry Science and Engineering, University of Lleida, Lleida, Spain; ^2^ Animal Health Department, Faculty of Veterinary Medicine, Universidad Complutense de Madrid, Madrid, Spain; ^3^ AIDS Research Group, August Pi i Sunyer Biomedical Research Institute (IDIBAPS), Hospital Clinic, University of Barcelona, Barcelona, Spain

**Keywords:** porcine reproductive and respiratory syndrome virus, broad neutralization, neutralizing antibody, cross-protection, *in vivo* protection

## Abstract

Porcine reproductive and respiratory syndrome (PRRS) is considered one of the most relevant diseases of swine. The condition is caused by PRRS virus (PRRSV), an extremely variable virus of the *Arteriviridae* family. Its heterogeneity can be responsible, at least partially, of the poor cross-protection observed between PRRSV isolates. Neutralizing antibodies (NAs), known to play a role in protection, usually poorly recognize heterologous PRRSV isolates, indicating that most NAs are strain-specific. However, some pigs develop broadly reactive NAs able to recognize a wide range of heterologous isolates. The aim of this study was to determine whether PRRSV isolates that induce broadly reactive NAs as determined *in vitro* are able to confer a better protection *in vivo*. For this purpose two *in vivo* experiments were performed. Initially, 40 pigs were immunized with a PRRSV-1 isolate known to induce broadly reactive NAs and 24 additional pigs were used as controls. On day 70 after immunization, the pigs were divided into eight groups composed by five immunized and three control pigs and exposed to one of the eight different heterologous PRRSV isolates used for the challenge. In the second experiment, the same experimental design was followed but the pigs were immunized with a PRRSV-1 isolate, which is known to generate mostly strain-specific NAs. Virological parameters, specifically viremia and the presence of challenge virus in tonsils, were used to determine protection. In the first experiment, sterilizing immunity was obtained in three groups, prevention of viremia was observed in two additional groups, although the challenge virus was detected occasionally in the tonsils of immunized pigs, and partial protection, understood as a reduction in the frequency of viremia compared with controls, was recorded in the remaining three groups. On the contrary, only partial protection was observed in all groups in the second experiment. The results obtained in this study confirm that PRRSV-1 isolates differ in their ability to induce cross-reactive NAs and, although other components of the immune response might have contributed to protection, pigs with cross-reactive NAs at the time of challenge exhibited better protection, indicating that broadly reactive NAs might play a role in protection against heterologous reinfections.

## Introduction

Porcine reproductive and respiratory syndrome (PRRS) remains one of the most important diseases of swine. The condition is characterized by reproductive failure in sows and boars and respiratory disease in growing pigs, leading to important economic losses in the vast majority of pork-producing countries (1). The etiological agent of PRRS is a small, enveloped, RNA virus, commonly known as porcine reproductive and respiratory syndrome virus (PRRSV), which has been recently classified in the genus *Betaarterivirus* within the family *Arteriviridae* in the order *Nidovirales* (2). The huge genomic variability of PRRSV isolates has led to their classification into two different species: *Betaarterivirus suid 1*, known as PRRSV-1, which predominates in European countries, and *Betaarterivirus suid 2*, known as PRRSV-2, widely spread in American and Asian countries (3). Even more, a significant genomic variability has been described within each species, and several subtypes and lineages have been identified (3–5). Genomic variability translates into high antigenic variability, which is considered as one of the main factors, which could explain the poor or, in the best case, the partial cross-protection observed upon heterologous virus exposure (6–9).

Although the key elements of the immune response against PRRSV involved in protection are not completely elucidated, both humoral and cell-mediated immunity seem to play a role (10). Thus, animals with a high proportion of PRRSV-specific IFN-γ secreting cells (IFN-γ SC) clear the virus after infection faster than animals with a poor cell-mediated immune response (11, 12) and the frequency of IFN-γ; SC is usually higher in protected pigs than in unprotected pigs (13). Even more, vaccinated and challenged pigs can be protected in the absence of neutralizing antibodies (NAs), supporting the notion that cell-mediated immunity can confer protection (14). Nevertheless, it is also well accepted that PRRSV-specific NAs play a role in protection, as it has been proven in passive transfer studies in which the sole presence of NAs has been sufficient to confer protection against reproductive failure, viremia, or even infection to naïve gestating sows (15) and to prevent viremia and even infection in growing pigs, although in a dose-dependent manner (16).

However, the development of PRRSV-specific NAs and IFN-γ SC is considered to be slow (10). Besides, and even more important, PRRSV variability can condition the ability of the previously generated immunity to recognize heterologous strains. As regards cell-mediated immunity, whereas some studies report broad cross-reactivity of PRRSV-specific IFN-γ SC (17), others indicate huge variability in the recognition of heterologous strains by IFN-γ SC (18, 19). This effect is more pronounced in the case of the humoral immune response, and particularly of NAs. Thus, several studies carried out with both PRRSV-1 and PRRSV-2 have demonstrated that NA cross-reactivity in seroneutralization (SN) assays is very limited, even in the case of closely related isolates, indicating that most NAs generated upon infection are strain-specific and do not recognize heterologous viruses (20–22).

Nonetheless, field and experimental evidences indicate that a small proportion of sera contain broadly reactive NAs able to recognize and neutralize heterologous viruses. Thus, in a recent immunization and challenge study, 1% of the vaccinated pigs developed NAs able to recognize efficiently six genetically diverse viruses in SN assays (22). In addition, a small proportion of field sera obtained from sows in PRRSV-unstable farms exhibited neutralizing activity against diverse PRRSV, including PRRSV-1 and PRRSV-2 isolates (23). Both studies indicate the positive effect of repeated exposures to heterologous viruses in the generation of broadly reactive NAs against PRRSV. Besides, there are studies that indicate the individual properties of the PRRSV to which the pigs are exposed, which might also play a role in the induction of broadly reactive NAs (21, 24). Yet, the proportion of PRRSV isolates able to induce broadly reactive NAs, at least in the case of PRRSV-1, seems to be very low. Thus, in a study carried out by Martinez-Lobo et al. (21), 29 PRRSV-1 monospecific hyperimmune sera were generated and confronted to a panel of 39 different PRRSV-1 isolates in SN assays. The results of that study indicate that only around 10% of the sera produced exhibited a significant level of cross-reactivity and were able to recognize and neutralize, at least to some extent, most of the viruses of the panel. Only three sera recognized all viruses with geometric mean of the titer (GMT) of NAs higher than 4 log_2_ (i.e., 1:16). On the contrary, up to 35% of the sera generated exhibited a GMT of NAs lower than 2 log_2_ and recognized poorly many of the isolates used in the SN assays. The ability to induce broadly reactive NAs might be relevant for the induction of a protective immune response. However, up to date, it is unclear whether cross-reactivity in SN assays carried out *in vitro* correlates with protection *in vivo*. As a consequence, the aim of this study was to determine whether PRRSV isolates that induce broadly reactive NAs *in vitro* are able to confer a better protection *in vivo* upon exposure to heterologous strains than PRRSV isolates that induce mostly strain-specific NAs.

## Materials and Methods

### Virus Isolates and Cell Cultures

Two PRRSV-1 isolates were selected for the immunization of pigs in this study. The selection of those viruses was based on the results of a previous study in which the cross-reactivity of NAs raised against a total of 29 individual PRRS-1 viruses was measured *in vitro* in SN assays against a panel of 39 viruses (21). The first isolate selected, Sp-3a, induced broadly reactive NAs in that study. The breadth of the monospecific sera generated was 100% (i.e., it was able to neutralize, at least to some extent, all viruses of the panel) and its potency is high, with a GMT of NAs of 6.17 log_2_. On the contrary, the second isolate selected, EU-11a, did not induce broadly reactive NAs. Its monospecific serum was able to recognize only 80% of the viruses with a GMT of NAs of 1.36 log_2_.

Both isolates were cultured and titrated in MARC-145, a cell clone derived from MA-104 cell line, highly permissive for PRRSV infection (25). MARC-145 cells were cultured in Dulbecco’s modified Eagle’s medium (DMEM) (Gibco, Invitrogen) supplemented with 10% fetal bovine serum (FBS) (Gibco, Invitrogen) and 1× antibiotic-antimycotic mixture (i.e., 100 IU penicillin/ml, 100 µg streptomycin/ml, and 0.25 µg fungizone/ml) (Gibco, Invitrogen) at 37°C in an atmosphere with 5% CO_2_. MARC-145 monolayers with approximately 90% confluency were infected with one of the selected PRRSV isolates and kept in culture until cytopathic effect (CPE) reached approximately 80% to 90%. Then, after three cycles of freezing and thawing, the cultures were clarified by centrifugation. Virus titers were determined as described by Scortti et al. (26). Viral titers were calculated following the method developed by Reed and Muench (27) and expressed as tissue culture infectious doses 50 (TCID_50_)/ml.

Nine different heterologous PRRSV isolates were selected for challenge (**Table 1**). Those viruses were selected on the basis of genetic distance of the genome region coding for major envelope proteins (**Table 2**) and on previous cross-reactivity results in SN assays (**Table 1**). PRRSV isolates used for challenge were cultured and titrated in primary cultures of porcine alveolar macrophages (PAMs) prepared as previously described (28), with the exception of PRRSV-2 AM-5 isolate that was propagated in MARC-145 cell cultures prepared as described above. However, when these viruses were used for SN assays, they were cultured in MARC-145 cell line as previously described. Only viruses exhibiting obvious CPE in MARC-145 cell line were used in this study.

**Table 1:** Description of the main characteristics of the PRRSV isolates used for challenge in Experiments A and B.

Experiment A						Experiment B					
Experimental Group	Challenge Isolate	Species	Country of isolation	Year of isolation	NA titer in cross-SN assay	Experimental Group	Challenge Isolate	Species	Country of isolation	Year of isolation	NA titer in cross-SN assay
A.1	EU-11	PRRSV-1	Czech Republic	1996	9^a^	B.1	Sp-3	PRRSV-1	Spain	1992	2^a^
A.2	AM-5	PRRSV-2	EE.UU.	1996	0.1	B.2	AM-5	PRRSV-2	EE.UU.	1996	0.1
A.3	EU-17	PRRSV-1	Italy	2004	0.1	B.3	EU-17	PRRSV-1	Italy	2004	0.1
A.4	Sp-27	PRRSV-1	Spain	2003	6	B.4	Sp-27	PRRSV-1	Spain	2003	0.1
A.5	EU-5	PRRSV-1	The Netherlands	1995	4	B.5	EU-5	PRRSV-1	The Netherlands	1995	4
A.6	EU-15	PRRSV-1	Italy	1995	5	B.6	EU-15	PRRSV-1	Italy	1995	0.1
A.7	Sp-22	PRRSV-1	Spain	2002	7	B.7	Sp-22	PRRSV-1	Spain	2002	3
A.8	Sp-29	PRRSV-1	Spain	2003	4	B.8	Sp-29	PRRSV-1	Spain	2003	0.1

a Results are expressed as log_2_ of the reciprocal of the serum dilution that completely inhibited viral replication in 50% of the wells. Values obtained from Martínez-Lobo et al. (21).

**Table 2:** Pairwise comparison of the nucleotide sequences of ORFs 2, 3, 4, 5, 6 and 2-6 of the PRRSV isolates used for immunization and challenge in Experiments A and B.

Challenge isolate	Immunization isolate Sp-3a						Challenge isolate	Immunization isolate EU-11a					
	ORF2	ORF3	ORF4	ORF5	ORF6	ORF2-6		ORF2	ORF3	ORF4	ORF5	ORF6	ORF2-6
AM-5	63.6	62.2	65.5	61.0	69.1	64.6	AM-5	60.0	62.0	64.8	61.6	69.1	64.3
EU-17	86.5	83.4	84.9	86.1	86.0	85.6	EU-17	82.8	85.2	85.1	85.4	87.5	86.2
Sp-27	89.2	85.9	92.0	90.0	97.5	91.6	Sp-27	83.4	84.4	90.9	89.2	97.3	90.4
EU-5	93.8	93.7	94.9	93.2	96.1	94.1	EU-5	90.0	97.8	97.8	97.6	99.2	97.0
EU-15	89.7	87.3	87.1	80.6	91.5	89.2	EU-15	84.5	88.5	87.1	80.3	90.0	88.9
Sp-22	91.0	90.6	90.9	92.4	97.1	92.1	Sp-22	86.4	90.4	89.8	89.4	95.9	91.1
Sp-29	90.6	85.8	85.5	90.5	93.6	89.7	Sp-29	86.0	89.5	86.9	89.4	93.6	90.3
EU-11	86.5	92.9	93.1	92.2	96.5	92.8	Sp-3	86.5	92.9	93.1	92.2	96.5	92.8

### Animals and Experimental Design

A total of one hundred and twenty-eight 10-week-old cross-bred pigs from a herd free of PRRSV and with no measurable PRRSV serum antibody titers were used in two different experiments. All experimental procedures were approved by the Animal Ethics Committee of Complutense University. In each experiment, 64 pigs were used. In experiment A, after an acclimatization period of 7 days, 40 randomly selected pigs were immunized by the intramuscular (IM) route three times 3 weeks apart with 10^5^ TCID_50_ of Sp-3a isolate, whereas the remaining 24 pigs were kept as controls. In experiment B, the same protocol was followed using EU-11a as immunization virus.

Four weeks after the last immunization, the pigs of each experiment were randomly divided into eight groups composed by five PRRSV-immunized and three control pigs and were housed in isolation. Then, the eight pigs of each group were exposed intranasally (IN) to 5 ml of the clarified supernatant of a PRRSV-infected PAM culture containing a total of 10^5^ TCID_50_ of one of the PRRSV isolates selected for challenge.

Every day, from 3 days before challenge until 10 days post-challenge, rectal temperatures were measured. In addition, clinical signs were evaluated daily following a previously published score (29).

Blood samples were collected in serum-clot vacuum tubes on the day of each immunization (i.e., D0, D21, and D42 of the experiment), the day of the challenge (i.e., D70 of the experiment), and every 3 days thereafter until D91 (i.e., day 21 after the challenge). Serum samples were stored at −80°C until used for virus detection and for the determination of homologous and heterologous PRRSV NA titers. Twenty-one days after the challenge (i.e., D91 of the experiment), all pigs were euthanized, and a complete necropsy was performed. At necropsy, lungs were collected and examined to evaluate the presence and severity of macroscopic lung lesions following a previously published score (30). Besides, tonsils were collected and stored at −80°C until used for virus isolation and viral RNA sequencing.

### Virus Isolation and Titration

Samples were processed as previously described (28) and used to inoculate monolayers of PAM or MARC-145 cells, depending on the isolate considered, in quadruplicate. After 90 min at 37°C to facilitate adsorption, the monolayers were washed with DMEM and fresh DMEM supplemented with 10% FBS and 3× antibiotic–antimycotic mixture was added. The cells were incubated for 6 days at 37°C, in a humidified atmosphere containing 5% CO_2_ and checked for CPE on days 4, 5, and 6 post-inoculation. As a positive control, strain 5710 was added to DMEM to a final concentration of 10^4^, 10^3^, and 10^2^ TCID_50_/ml (i.e., 10^3^, 10^2^, and 10 TCID_50_/well). Only batches of PAM with a minimum sensitivity to infection of at least 50% of the wells, to which 10 TCID_50_ were added, were used. Virus-free DMEM or FBS were used as negative controls. If CPE was observed, reverse transcription polymerase chain reaction (RT-PCR) was carried out to confirm the presence of PRRSV-1 (31) or PRRSV-2 (32). Virus titers were determined as described by Scortti et al. (26).Titers were calculated using the method developed by Reed and Muench (27) and expressed as TCID_50_/g for tonsil samples or TCID_50_/mL for serum samples.

### PRRSV Sequencing

For determining whether the virus isolated from tonsils was the immunization or the challenge PRRSV isolate, ORF5 from positive samples was amplified and sequenced. For that purpose, total RNA was obtained using QIAmp^®^ Viral RNA Mini kit (Qiagen) following the manufacturer’s instructions. Then, PRRSV ORF5 was amplified by RT-PCR using a commercial kit (SuperScript III OneStep RT-PCR platinum TaqHiFi^®^, Invitrogen), following the manufacturer’s instructions. For this purpose, two different pairs of primers were used, one to amplify all PRRSV-1 viruses and another one to amplify the PRRSV-2 isolate used in the study (31, 32). RT-PCR products were purified using a commercial kit (QIAQuick^®^ Purification Gel Kit, Qiagen) following the manufacturer’s instructions. Individual sequences of both strands of the PCR products were determined with the same pair of primers used for RT-PCR, amplifying the samples by asymmetric PCR with fluorescent terminators and analyzing the products by electrophoresis on an ABI prism 310 Genetic Analyzer (Applied Biosystems). Sequences were manually corrected, purged of errors, and a bioinformatics analysis was performed. Sequences were aligned using Clustal Omega software (33).

### Serological Assays

PRRSV-specific antibodies in serum samples were determined using a commercial ELISA test (HerdChek PRRS ELISA 2XR, IDEXX Laboratories, Inc.).

To carry out SN assays, all sera were heat inactivated at 56°C for 30 min. Then two-fold dilutions of each serum were prepared using DMEM as diluent in 96-well tissue culture plates. Then 50 µl of a viral suspension of the same stock used for the immunization or for challenge containing 100 TCID_50_ of the appropriate PRRSV isolate prepared in DMEM were added to 50 µl of each serum dilution. Serum–viral mixtures were incubated for 1 h at 37°C in a humidified atmosphere containing 5% CO_2_. Thereafter, 100 µl of a cell suspension containing 1 ×10^4^ MARC-145 cells/well in DMEM supplemented with 10% heat inactivated FBS was added to the plates that were incubated at 37°C in a humidified atmosphere containing 5% CO_2_ for 6 days. All samples were analyzed in duplicate. The culture plates were examined for CPE on days 4, 5, and 6 post-inoculation. The titers were expressed as log_2_ of the reciprocal of the serum dilution that completely inhibited viral replication in 50% of the wells.

### Statistical Analysis

The occurrence of clinical signs and macroscopic lung lesions were evaluated for significance using Kruskal–Wallis non-parametric and Mann–Whitney U tests. A Student’s t test was used to assess significance in differences in rectal temperature before and after the challenge. GMT of NAs against the viruses used for immunization and challenge was estimated. Differences in GMT of NAs obtained for the different isolates were analyzed using Kruskal-Wallis non parametric and Mann-Whitney *U* tests. Differences in viral titers were evaluated for significance by one-way analysis of variance and Duncan’s multiple range test. In addition, differences in the proportion of positive samples were assessed for significance by the two-tailed Fisher’s exact test. Finally, the existence of a booster in NAs upon challenge was analyzed for significance by using Friedman and Dunn’s multiple comparison tests. All statistical tests were carried out with GraphPad Prism software, and results were considered as statistically significant when p value is less than 0.05.

## Results

### Serological Response to Immunization

On D0, all pigs were seronegative for PRRSV-specific systemic IgGs measured by ELISA test. However, from D21 to the end of the study, all animals remained seropositive in both experiments.

**Figure 1:**
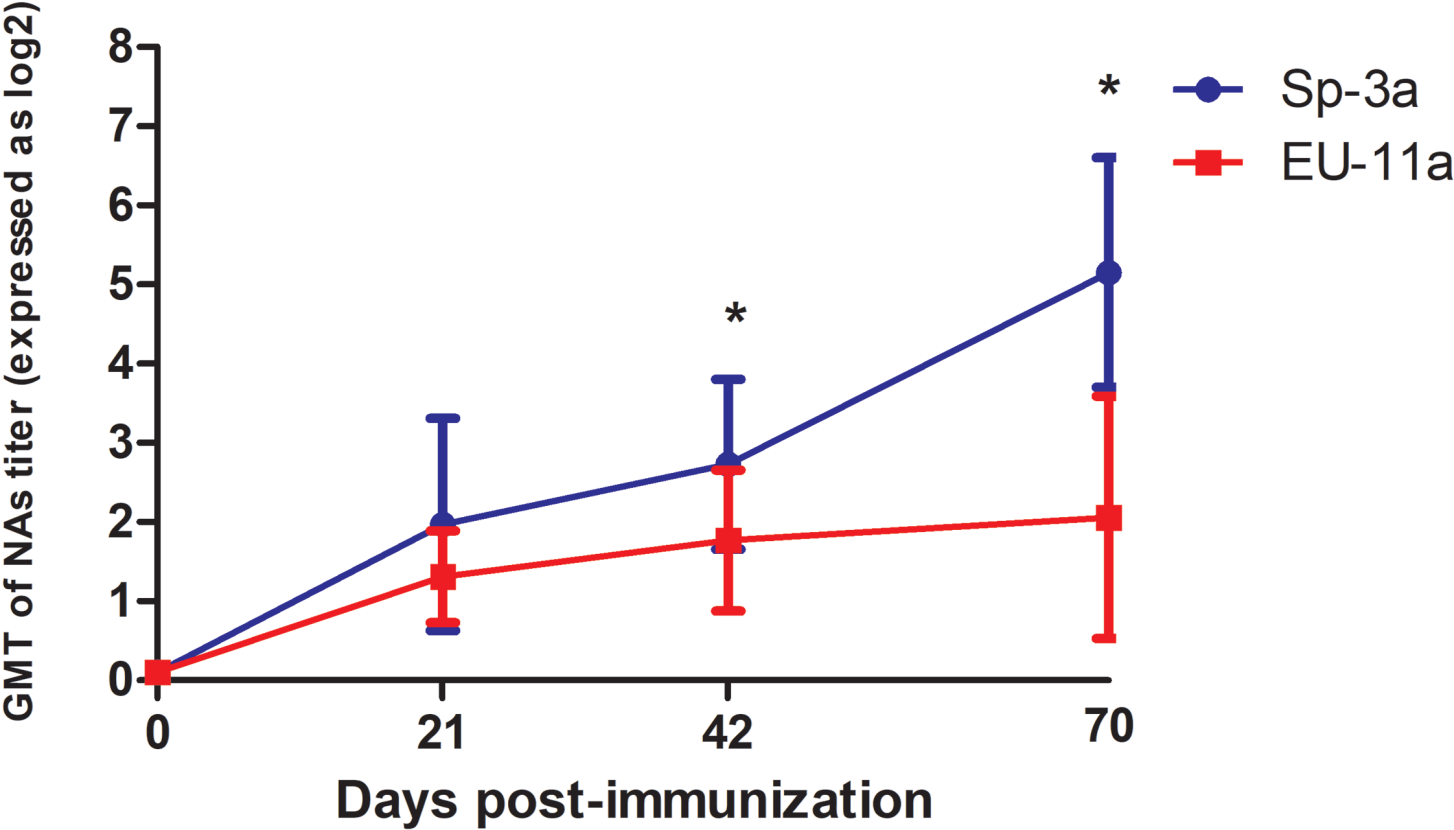
Dynamics of development of NAs against the PRRSV isolate used for immunization in Experiment A and Experiment B. Statistically significant differences (p < 0.05) are marked with a * symbol.

In the same way, no NAs were detected on the day of the first immunization in any of the pigs. However, on D21, most of the animals had detectable, although generally low, titers of NAs against the immunization PRRSV isolate, with GMTs of NAs of 1.97 log_2_ in the case of Sp-3a (i.e., Experiment A) and 1.31 log_2_ in the case of EU-11a (i.e., Experiment B) (**Figure 1**). At the time of the third immunization (i.e., D42), homologous NA titers have raised slightly and steadily in both experiments (A and B), reaching GMTs of 2.73 log_2_ and 1.77 log_2_ for Sp-3a and EU-11a, respectively. However, on the day of challenge (i.e., D70), in experiment A, the GMT of NAs against Sp-3a has raised sharply (i.e., GMT of 5.15 log_2_), whereas in the case of experiment B, the GMT of NAs against EU-11a have increased only moderately (i.e. GMT of 2.06 log_2_). The differences in NA titers against the immunization PRRSV isolate on the day the third immunization (i.e. D42) and the day of challenge (i.e. D70) were statistically significant (p < 0.05) between the experiments.

### Evaluation of Clinical Signs and Lesions

No remarkable clinical signs were observed after the challenge in any of the pigs, regardless if they were immunized or control pigs, neither in experiment A nor in experiment B. In the same way, rectal temperatures remained, with very few exceptions, within normal limits for pigs of that age after the challenge, and no statistically significant differences were observed between groups, between immunized and controls, or before and after viral exposure. Finally, no macroscopic lung lesions were observed at necropsy in any of the pigs, regardless if they were immunized or control pigs or the virus used for the challenge, neither in experiment A nor in experiment B.

In experiment B, one of the immunized pigs died on D56 of the experiment because of an incarcerated umbilical hernia. Consequently, only four immunized pigs were assigned to group B.7 at the time of the challenge.

### PRRSV Detection in Clinical Samples

All pigs were negative to PRRSV in serum samples on the day of the first immunization (i.e., D0 of the experiment). Then, on D21 (i.e., the day of the second immunization), all serum samples obtained from immunized pigs in both experiments (A and B) were positive by virus isolation. However, on D42 (i.e., the day of the third immunization), no virus was detected in any serum samples.

The results of the isolation of PRRSV from the serum samples obtained from the previously immunized and control pigs in experiments A and B on the day of challenge (i.e., D70) and afterward are shown in **Tables 3.1**, **3.2**, **4.1** and **4.2**. As can be seen in **Tables 3.1**, **3.2**, in experiment A (i.e. pigs immunized with PRRSV isolate Sp-3a), viremia could not be detected at any time after the challenge in any of the immunized pigs in five of the eight experimental groups. Conversely, viremia was detected in the immunized pigs of the other three experimental groups (i.e., groups A.2, A.3, and A.4). However, the frequency of detection was much lower than that in the control pigs and in most cases limited to the first few days after challenge and/or to some pigs. On the contrary, PRRSV was detected in serum samples from all control pigs from all experimental groups on most sampling days. Differences in the frequency of viremia between immunized and control pigs were statistically significant from day 3 post-challenge in all groups in which viremia was never detected in immunized pigs at any time post-challenge (p < 0.05). In addition, differences were also statistically significant in group A.2 from day 6 to day 18 post-challenge, in group A.4 from day 9 to day 21 post-challenge, and in group A.3 on day 9 post-challenge (p < 0.05). Conversely, no statistically significant differences were detected in the viral titer in serum samples between immunized and control pigs with the only exception of pigs of group A.3 on day 3 post-challenge (1.70 TCID_50_/ml in immunized pigs *versus* 3.29 TCID_50_/mL in controls, p < 0.05).

**Table 3.1:** Virus isolation from serum samples collected in Experiment A (pigs immunized with Sp-3a).

Days post-challenge	Group A.1 (EU-11)		Group A.2 (AM-5)		Group A.3 (EU-17)		Group A.4 (Sp-27)	
	Immunized	Controls	Immunized	Controls	Immunized	Controls	Immunized	Controls
D0	0/5 (-)*	0/3 (-)	0/5 (-)	0/3 (-)	0/5 (-)	0/3 (-)	0/5 (-)	0/3 (-)
D3	0/5^a^ (-)	3/3 (1.89 ± 0.54)	1/5 (1.66)	3/3 (1.67 ± 0.29)	4/5 (1.70 ± 0.09)^b^	3/3 (3.29 ± 0.06)	4/5 (1.67 ± 0.24)	3/3 (2.28 ± 0.63)
D6	0/5^a^ (-)	3/3 (2.11 ± 0.54)	0/5^a^ (-)	3/3 (1.67 ± 0.29)	1/5 (3.23)	2/2^c^ (3.05 ± 0.55)	2/5 (2.00 ± 0.71)	3/3 (3.05 ± 0.42)
D9	0/5^a^ (-)	3/3 (1.55 ± 0.09)	0/5^a^ (-)	3/3 (1.55 ± 0.09)	0/5^a^ (-)	3/3 (2.70 ± 0.67)	0/5^a^ (-)	3/3 (2.72 ± 0.68)
D12	0/5^a^ (-)	3/3 (1.50 ± 0.00)	0/5^a^ (-)	3/3 (1.83 ± 0.58)	1/5 (1.66)	3/3 (1.89 ± 0.54	0/5^a^ (-)	3/3 (2.28 ± 0.25
D15	0/5^a^ (-)	3/3 (1.50 ± 0.00)	0/5^a^ (-)	3/3 (2.00 ± 0.50)	1/5 (1.50)	3/3 (2.27 ± 1.20)	0/5^a^ (-)	3/3 (2.22 ± 0.48)
D18	0/5^a^ (-)	3/3 (1.55 ± 0.09)	0/5^a^ (-)	3/3 (1.55 ± 0.09)	1/5 (1.66)	3/3 (2.72 ± 0.85)	0/5^a^ (-)	3/3 (1.67 ± 0.29)
D21	0/5^a^ (-)	3/3 (1.50 ± 0.00)	1/5 (1.50)	3/3 (1.78 ± 0.48)	1/5 (1.66)	3/3 (1.80 ± 0.38)	0/5^a^ (-)	3/3 (1.67 ± 0.29)

*Frequency of isolation of PRRSV from serum samples. Mean titer and standard deviation are indicated in parentheses.

^a^Indicates statistically significant differences in the frequency of isolation between the immunized and control pigs.

^b^Indicates statistically significant differences in the viral titers in serum samples between the immunized and control pigs.

^c^The serum sample of one of the control pigs was not available on day 6 post-challenge.

**Table 3.2:** Virus isolation from serum samples collected in Experiment A (pigs immunized with Sp-3a).

Days post-challenge	Group A.5. (EU-5)		Group A.6. (EU-15)		Group A.7. (Sp-22)		Group A.8. (Sp-29)	
	Immunized	Controls	Immunized	Controls	Immunized	Controls	Immunized	Controls
D0	0/5 (-)*	0/3 (-)	0/5 (-)	0/3 (-)	0/5 (-)	0/3 (-)	0/5 (-)	0/3 (-)
D3	0/5^a^ (-)	3/3 (2.33 ± 0.33)	0/5^a^ (-)	3/3 (2.24 ± 0.37)	0/5^a^ (-)	3/3 (3.72 ± 0.26)	0/5^a^ (-)	3/3 (3.39 ± 0.79)
D6	0/5^a^ (-)	3/3 (1.83 ± 0.44)	0/5^a^ (-)	3/3 (2.78 ± 0.63)	0/5^a^ (-)	3/3 (2.22 ± 0.38)	0/5^a^ (-)	3/3 (2.61 ± 0.09)
D9	0/5^a^ (-)	3/3 (2.22 ± 0.48)	0/5^a^ (-)	3/3 (1.78 ± 0.48)	0/5^a^ (-)	3/3 (2.16 ± 0.44)	0/5^a^ (-)	3/3 (2.22 ± 0.35)
D12	0/5^a^ (-)	3/3 (1.67 ± 0.29)	0/5^a^ (-)	3/3 (2.16 ± 0.01)	0/5^a^ (-)	3/3 (1.50 ± 0.00)	0/5^a^ (-)	3/3 (2.00 ± 0.50)
D15	0/5^a^ (-)	3/3 (1.77 ± 0.20)	0/5^a^ (-)	3/3 (1.83 ± 0.29)	0/5^a^ (-)	3/3 (1.50 ± 0.00)	0/5^a^ (-)	2/3 (2.00 ± 0.71)
D18	0/5^a^ (-)	3/3 (1.94 ± 0.63)	0/5^a^ (-)	3/3 (1.55 ± 0.09)	0/5^a^ (-)	3/3 (1.55 ± 0.09)	0/5^a^ (-)	3/3 (1.83 ± 0.29)
D21	0/5^a^ (-)	3/3 (1.50 ± 0.00)	0/5^a^ (-)	3/3 (1.50 ± 0.00)	0/5^a^ (-)	3/3 (1.74 ± 0.42)	0/5^a^ (-)	3/3 (1.83 ± 0.58)

*Frequency of isolation of PRRSV from serum samples. Mean titer and standard deviation are indicated in parentheses.

^a^Indicates statistically significant differences in the frequency of isolation between the immunized and control pigs.

**Table 4.1:** Virus isolation from serum samples collected in Experiment B (pigs immunized with EU-11a).

Days post-challenge	Group B.1. (Sp-3)		Group B.2. (AM-5)		Group B.3. (EU-17)		Group B.4. (Sp-27)	
	Immunized	Controls	Immunized	Controls	Immunized	Controls	Immunized	Controls
D0	0/5 (-)*	0/3 (-)	0/5 (-)	0/3 (-)	0/5 (-)	0/3 (-)	0/5 (-)	0/3 (-)
D3	3/5 (2.05 ± 0.52)	3/3 (2.39 ± 0.34)	5/5 (2.31 ± 0.59)	3/3 (2.37 ± 0.90)	5/5 (2.76 ± 1.05)	2/3 (2.50 ± 0.17)	4/5 (1.86 ± 0.61)	3/3 (1.53 ± 0.03)
D6	3/5 (2.22 ± 0.51)	3/3 (2.61 ± 0.92)	5/5 (2.10 ± 0.55)^b^	3/3 (3.67 ± 0.52)	5/5 (3.43 ± 1.00)	3/3 (3.05 ± 0.58)	5/5 (2.03 ± 0.91)	3/3 (2.83 ± 1.44)
D9	1/5 (2.00)	3/3 (2.11 ± 0.51)	4/5 (1.65 ± 0.57)	3/3 (2.83 ± 0.26)	4/5 (3.35 ± 0.90)	3/3 (3.22 ± 0.38)	4/5 (2.54 ± 0.71)	3/3 (2.72 ± 1.11)
D12	4/5 (1.83 ± 0.20)	3/3 (1.89 ± 0.067)	5/5 (2.20 ± 0.45)	3/3 (2.17 ± 0.05)	4/5 (2.79 ± 0.91)	3/3 (2.27 ± 1.20)	3/5 (1.72 ± 0.26)	3/3 (2.78 ± 1.11)
D15	4/5 (2.00 ± 0.71)	3/3 (2.28 ±.0.75)	5/5 (1.50 ± 0.00)	3/3 (1.80 ± 0.52)	4/5 (2.21 ± 0.95)	3/3 (2.55 ± 1.02)	2/5 (3.00 ± 0.94)	3/3 (2.24 ± 0.64)
D18	2/5 (3.00 ± 2.05)	3/3 (1.88 ± 0.39)	4/5 (1.50 ± 0.00)	3/3 (1.55 ± 0.09)	4/5 (1.87 ± 0.53)	3/3 (2.33 ± 0.76)	1/5 (1.50)	3/3 (2.44 ± 1.63)
D21	2/5 (1.50 ± 0.00)	2/3 (2.42 ± 1.29)	2/5 (1.57 ± 1.17)	2/3 (1.50 ± 0.00)	3/5 (1.94 ± 0.42)	2/3 (2.00 ± 0.71)	1/5 (2.66)	2/3 (2.11 ± 0.63)

*Frequency of isolation of PRRSV from serum samples. Mean titer and standard deviation are indicated in parentheses.

^b^Indicates statistically significant differences in the viral titers in serum samples between the immunized and control pigs.

**Table 4.2:** Virus isolation from serum samples collected in Experiment B (pigs immunized with EU-11a).

Days post-challenge	Group B.5. (EU-5)		Group B.6. (EU-15)		Group B.7. (Sp-22)		Group B.8. (Sp-29)	
	Immunized	Controls	Immunized	Controls	Immunized	Controls	Immunized	Controls
D0	0/5 (-)*	0/3 (-)	0/5 (-)	0/3 (-)	0/5 (-)	0/3 (-)	0/5 (-)	0/3 (-)
D3	0/5^a^ (-)	3/3 (1.55 ± 0.09)	5/5 (1.66 ± 0.20)	3/3 (1.55 ± 0.09)	4/4 (2.68 ± 0.33)	3/3 (2.61 ± 0.09)	4/5 (2.53 ± 0.66)	3/3 (2.22 ± 0.63)
D6	1/5 (1.50)	3/3 (3.11 ± 0.51)	3/5 (2.39 ± 0.34)	3/3 (3.22 ± 0.38)	3/4 (2.27± 0.67)	3/3 (3.73 ± 0.77)	0/5^a^ (-)	3/3 (3.84 ± 0.43)
D9	2/5 (1.50)	3/3 (2.05 ± 0.58)	2/5 (2.33 ± 0.00)	3/3 (2.22 ± 0.63)	3/4 (1.50 ± 0.00)	3/3 (3.29 ± 0.55)	1/5 (1.66)	3/3 (2.73 ± 0.77)
D12	4/5 (2.12± 0.69)	3/3 (2.00 ± 0.34)	3/5 (2.27 ± 0.67)	3/3 (2.61 ± 0.35)	2/4 (1.58 ± 0.11)	3/3 (2.77 ± 0.20)	4/5 (1.83 ± 0.56)^b^	3/3 (3.83 ± 0.58)
D15	1/5 (1.50)	2/3 (2.25 ± 1.06)	3/5 (2.00 ± 0.87)	3/3 (2.00 ± 0.34)	4/4 (2.37 ± 0.80)	3/3 (2.55 ± 1.54)	2/5 (1.58 ± 0.11)	3/3 (2.61 ± 0.35)
D18	0/5^a^ (-)	2/3 (2.17 ± 0.23)	3/5 (2.33 ± 1.04)	3/3 (2.33 ± 0.33)	2/4 (2.08 ± 0.59)	3/3 (1.99 ± 0.58)	1/5 (4.00)	3/3 (2.05 ± 0.42)
D21	0/5^a^ (-)	2/3 (1.83 ± 0.24)	2/5 (1.92 ± 0.59)	2/3 (1.75 ± 0.12)	2/4 (1.83 ± 0.24)	3/3 (1.67 ± 0.29)	1/5 (2.00)	1/3 (2.33 ± NA)

*Frequency of isolation of PRRSV from serum samples. Mean titer and standard deviation are indicated in parentheses.

^a^Indicates statistically significant differences in the frequency of isolation between the immunized and control pigs.

^b^Indicates statistically significant differences in the viral titers in serum samples between the immunized and control pigs.

In contrast, protection of pigs previously immunized with PRRSV-1 EU-11a in experiment B can be considered very poor. Thus, viremia was frequently detected at most time-points after challenge in immunized pigs (**Tables 4.1**, **4.2**). In the same way, control pigs show a pattern of viremia similar to that observed in experiment A, with most pigs positive at all sampling days. The differences in the proportion of viremic pigs between immunized and control pigs were not statistically significant with the exception of group B.5 on days 3, 18, and 21 post-challenge and group B.8 on day 6 post-challenge (p < 0.05). In the same way, viral titers in viremic pigs were similar between immunized and controls except in group B.2 on day 6 post-challenge and in group B.8 on day 12 post-challenge (p < 0.05).

Viral load in blood samples of controls pigs was compared between experiments. Although globally viral titers tended to be higher in experiment B, differences were statistically significant only in pigs exposed to AM-5 and Sp-22 (p < 0.05). Nonetheless, when viral titers were compared day by day, viral titers were significantly higher in experiment A in some days and in experiment B in some others (data not shown).

The results of PRRSV detection and identification in tonsils in experiments A and B are summarized in **Tables 5.1**, **5.2**. As it can be observed in the tables, PRRSV could be detected in the tonsils of a variable number of immunized pigs in all groups. However, sequencing of the viruses identified indicated that in three of the eight experimental groups (i.e., groups A.1, A.5, and A.6), the virus present in tonsils was the immunization isolate, whereas in the other five groups, the virus present in tonsils was the challenge virus (i.e., groups A.2, A3, A.4, A.7, and A.8). As expected, the challenge PRRSV was found in the tonsils of most of the previously immunized pigs of the groups in which the viremia was detected. However, and remarkably, the challenge virus was also found in one immunized pig of group A.8 and in two immunized pigs of group A.7, in which viremia was never recorded, suggesting that protection achieved in those animals was not sterilizing.

**Table 5.1:** Results of PRRSV RT-PCR and PRRSV isolate identification by sequencing from tonsils collected at necropsy from pigs in Experiment A.

Experiment A	Group A.1 (EU-11)	Group A.2 (AM-5)	Group A.3 (EU-17)	Group A.4 (Sp-27)	Group A.5 (EU-5)	Group A.6 (EU-15)	Group A.7 (Sp-22)	Group A.8 (Sp-29)
RT-PCR positive samples (%)	5/5 (100)	5/5 (100)	5/5 (100)	4/5 (80)	4/5 (80)	5/5 (100)	4/5 (80)	4/5 (80)
Immunization virus (%)	5/5 (100)	0/5 (0)	0/5 (0)	1/5 (20)	4/5 (80)	5/5 (100)	2/5 (40)	3/5 (60)
Challenge virus (%)	0/5 (0)	5/5 (100)	5/5 (100)	3/5 (60)	0/5 (0)	0/5 (0)	2/5 (40)	1/5 (20)

**Table 5.2:** Results of PRRSV RT-PCR and PRRSV isolate identification by sequencing from tonsils collected at necropsy from pigs in Experiment B.

Experiment A	Group B.1 (Sp-3)	Group B.2 (AM-5)	Group B.3 (EU-17)	Group B.4 (Sp-27)	Group B.5 (EU-5)	Group B.6 (EU-15)	Group B.7 (Sp-22)	Group B.8 (Sp-29)
RT-PCR positive samples (%)	5/5 (100)	5/5 (100)	5/5 (100)	5/5 (100)	5/5 (100)	4/5 (80)	4/4 (100)	4/5 (80)
Immunization virus (%)	0/5 (0)	0/5 (0)	0/5 (0)	0/5 (0)	0/5 (0)	0/5 (0)	0/4 (0)	0/5 (0)
Challenge virus (%)	5/5 (100)	5/5 (100)	5/5 (100)	5/5 (100)	5/5 (100)	4/5 (80)	4/4 (100)	4/5 (80)

On the contrary, most of the tonsils obtained from pigs immunized with EU-11a in experiment B were positive for PRRSV challenge isolate.

Finally, PRRSV was found in the tonsils of all control pigs, regardless the study considered or the group they belonged to.

### Cross-Reactivity of NAs at the Time of Challenge and Evolution of Homologous and Heterologous NAs

**Figure 2:**
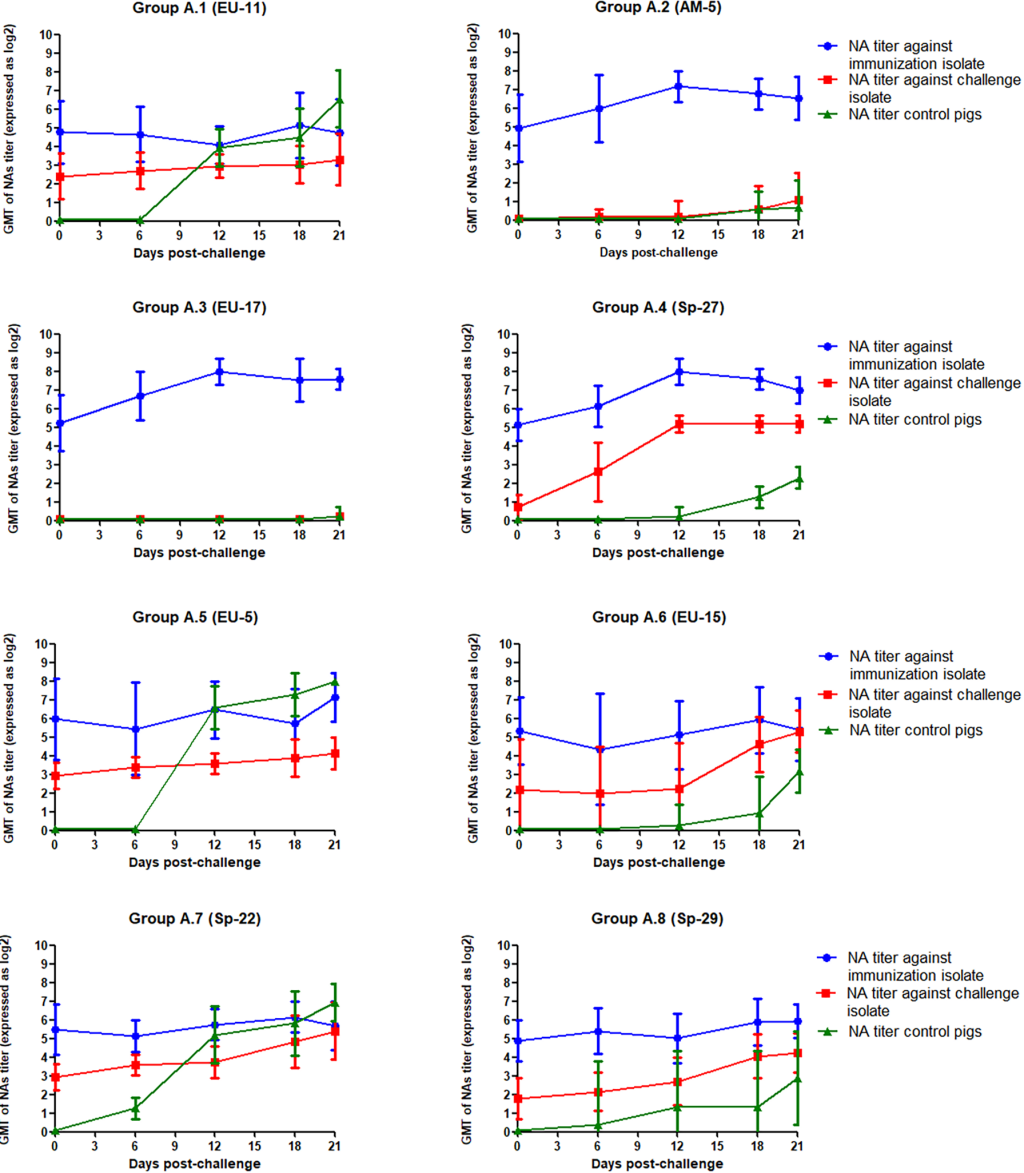
GMT of NAs against the immunization and challenge PRRSV isolates in immunized pigs and against challenge PRRSV isolate in control pigs in the eight groups included in the study from the day of challenge to the end of the experiment in Experiment A. The graph illustrates the GMT ± standard deviation.

The GMT of NAs against the PRRSV isolates used for immunization and challenge for each group in experiment A is depicted in **Figure 2**. As it can be observed in the figure, GMT of NAs against Sp-3a was quite similar between experimental groups the day of the challenge and ranged from 4.77 log_2_ in group A.1 to 5.96 log_2_ in group A.5. However, the GMT of NAs against the PRRSV isolate used for the challenge was more variable and ranked from undetectable levels in groups A.2 and A.3 to 2.93 log_2_ in groups A.5 and A.7. Then, after the challenge, the GMT of NAs against Sp-3a remained relatively steady in the groups in which viremia was not observed after challenge, whereas in groups A.2, A.3, and A.4, in which viremia was recorded, a slight increase in the GMT of NAs against Sp-3a was observed. The differences in NA titers immediately before and after challenge were statistically significant in groups A.3 and A.4, but not in group A.2 (p < 0.05). Regarding the response of NAs against the PRRSV isolate used for challenge, remarkable differences were noted between groups. Thus, in some groups NAs were barely detectable during the whole experimental period (i.e., groups A.2 and A.3), in some other groups, the titers of NAs remained relatively stable (i.e., groups A.1 and A.5), and in some others, an increased in GMT of NAs was recorded (i.e., groups A.4, A.6, A.7, and A.8). Nonetheless, differences in NA titers before and after challenge were statistically significant only in the case of group A.4 (p < 0.05).

**Figure 3:**
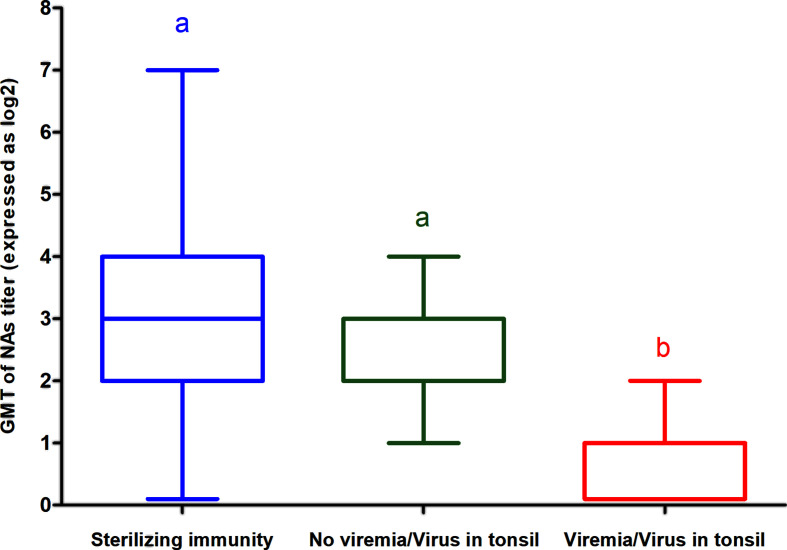
GMT of NAs against the challenge virus on D70 of the experiment (i.e day of challenge) in groups in which sterilizing immunity was observed, groups in which viremia was not detected but the challenge virus was occasionally detected in tonsils and groups in which viremia was detected upon challenge of immunized pigs in experiment A. Each box represents 25–75% of observation. Whiskers above and below of each box represent the lowest datum still within 1.5 interquartile range (IQR) of the lower quartile, and the highest datum still within 1.5 IQR of the upper quartile. Solid line within each box is the median. Statistically significant differences are highlighted by different letters.

When the GMT of NAs against the challenge virus on D70 of the experiment (i.e., day of challenge) was compared between groups in which the challenge virus was never found in immunized pigs (i.e., groups in which sterilizing immunity was observed), groups in which viremia was not detected but the challenge virus was occasionally detected in tonsils and groups in which viremia was detected upon challenge of immunized pigs, it was observed that titers were higher in the groups with sterilizing immunity than in groups in which the challenge virus was detected in tonsils and both titers were higher than in groups in which viremia was detected in immunized pigs. However, differences were statistically significant only between the groups in which viremia was detected and the groups in which viremia was never detected, regardless whether challenge virus could be detected in tonsils or not (**Figure 3**).

**Figure 4:**
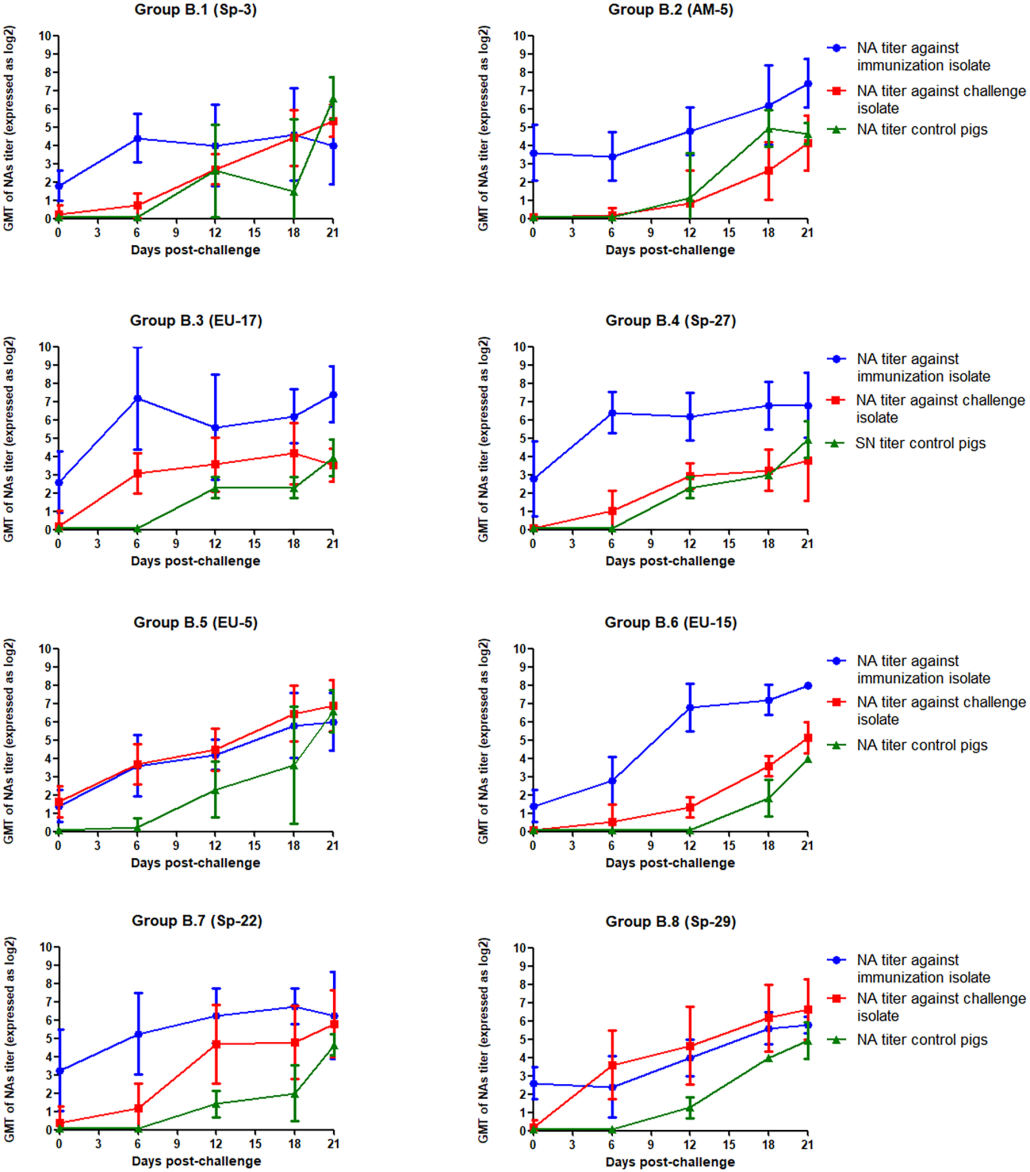
GMT of NAs against the immunization and challenge PRRSV isolates in immunized pigs and against challenge PRRSV isolate in control pigs in the eight groups included in the study from the day of challenge to the end of the experiment in experiment B. The graph illustrates the GMT ± standard deviation.

The GMT of NAs against PRRSV isolates used for immunization and challenge for each group in experiment B is depicted in **Figure 4**. In this case, GMT of NAs against EU-11a were lower than those recorded against Sp-3a in experiment A and ranged from 1.40 log_2_ in groups B.5 and B.6 to 3.60 log_2_ in group B.2. However, NAs against the challenge isolates were undetectable in most cases, and only in group B.5 reached values close to 2 log_2_. Then, after challenge, and contrary to what happened in experiment A, the GMT of NAs against EU-11a increased in all experimental groups, with statistically significant differences (p < 0.05), with the only exception of group B.7. The same phenomenon was observed in the case of the GMT of NAs against the viruses used for challenge, and in this case, differences were statistically significant in all experimental groups (p < 0.05).

## Discussion

Although PRRSV infection is considered to be long-lasting, infected pigs eventually clear the virus (34), which indicates that they are capable of mounting an immune response effective in the resolution of the infection (10). Even more, this immune response seems to be effective in the prevention of homologous reinfections (35) and, as least partially, in the prevention of heterologous infections (7, 8, 36). In this protective immunity, both cell-mediated and NAs are considered to play a role (14).

However, and despite the crucial role that NAs may play in the prevention of reinfections, their efficacy is largely compromised by their generally accepted lack of cross-reactivity. Nonetheless, in the last few years, the existence of broadly reactive NAs able to recognize a significant number of genetically diverse PRRSV has been reported. The generation of these broadly reactive NAs can be stimulated by multiple exposures to different viruses over time (9, 23, 37) or derive from repeated exposures to the same PRRSV isolate (21).

Our hypothesis in the present study was that individual viral properties might play a role in the induction of broadly reactive NAs and that those broadly reactive NAs might confer better protection against reinfections. To test this hypothesis, we selected two different viruses characterized in a previous study. The first virus, Sp-3a, induces broadly reactive NAs, whereas the second one, EU-11a, is a virus that induces mostly monospecific NAs with a very limited cross-reactivity. These two viruses were used in two *in vivo* experiments designed to optimize the generation of NAs and to test the protection of the immunized pigs against PRRSV heterologous challenges.

As this study was designed as a proof-of-concept to determine the value of NAs for protection, three immunizations were carried out in 10-week-old pigs, rather than using the regular 3-week-old pig model and a single viral exposure. We selected the pigs at the beginning of the growing period because, in our experience, older pigs tend to develop higher titers of NAs than younger pigs. Besides, we decided to follow the same protocol of immunization used in our previous *in vitro* study, to be able to compare *in vitro* and *in vivo* results. Consequently, we gave the pigs three immunizations 3 weeks apart. These repeated exposures were intended to mimic a hyperimmunization protocol and, theoretically, could have increased the chances of generating broadly reactive NAs in stimulating affinity maturation through hypersomatic mutation of antibodies directed against conserved neutralizing epitopes, as it has been previously suggested (23). Finally, 4 weeks after the last immunization, the pigs were exposed to one of the eight heterologous PRRSV isolate used in the study.

The protocol followed for immunization and the age of the pig at the beginning of the study led to the pigs being challenged at the age of 140 days. In our experience, experimental exposure of pigs to PRRSV-1 under controlled conditions at that age does not produce measurable clinical signs, negative effects on productive parameters or remarkable lung lesions: The same phenomenon has recently been described for PRRSV-2 viruses (12). Thus, as expected, none of the pigs, neither immunized nor controls, showed any remarkable clinical signs in any of the two experiments. In the same way, rectal temperatures remained, with very few exceptions, within normal limits for pigs of their age during the post-challenge period, and no macroscopic lesions were observed in the lungs at necropsy. As a consequence, the level of protection upon challenge was estimated using virological parameters, specifically the development of viremia and the establishment of a carrier state in the tonsils at the end of the experiments. Those virological parameters were selected because they are considered the most reliable indicators of protection in pigs of that age (12). The virological results were compared between immunized and control pigs and between experiments. In these comparisons, an unexpected finding was that viral load in blood samples was higher in experiment B. Although it can be speculated that these results indicate a higher susceptibility to infection of the pigs used in experiment B, all animals were purchased from the same farm and had the same genetic background and were randomly distributed between groups. Besides, when all data were analyzed together, differences in viral titer were statistically significant only in two groups (i.e., pigs challenged with AM-5 and with Sp-22). No statistically significant differences were found between control groups in the remaining six challenge viruses, and when differences were analyzed day by day, additional statistically significant differences were detected but sometimes favored the pigs of experiment A and some others pigs of experiment B (data not shown). Thus, a general pattern could not be established, and we believe that these differences might be related to the low number of pigs used per control group and that they do not affect significantly to the general results of the study and their interpretation.

The results obtained indicate that pigs initially immunized with PRRSV isolate Sp-3a achieve a remarkable level of protection upon a secondary exposure to a heterologous PRRSV. Thus, in three of the eight groups in experiment A, immunity developed upon immunization was considered sterilizing as no virus was found in serum samples or in tonsils at necropsy. The achievement of sterilizing immunity against a heterologous exposure is an extremely rare event in the case of highly variable RNA viruses and, to our knowledge, it has never been reported for PRRSV-1. On the contrary, although extremely rare as well, a remarkable level of protection has been recently described with a centralized immunogen developed using full-genome sequences of PRRSV-2 (24). Both studies confirm that very high levels of heterologous protection are possible against both PRRSV species.

Protection was also notable in two additional experimental groups in experiment A in which viremia was never detected upon heterologous challenge. Nonetheless, in those groups, immunity was not sterilizing, and the challenge virus was detected in the tonsils of some pigs. This same phenomenon has been observed in the study by López et al. (16) in which NAs titers from 1:8 to 1:32 were passively transferred to 2-week-old piglets before they were challenged. We do not know how the heterologous viruses used in our study reached the tonsils despite being never detected in serum, but several explanations are possible. One possibility is that viremia might have been established but the maximum viral load reached was below the detection threshold of the technique used in our study. We selected virus isolation for PRRSV detection to make sure that we detected only viable virus, but the downside of this approach is that the sensitivity of detection is lower than that of RT-qPCR technique. Nonetheless, previous studies show that although the frequency of viremia is lower in vaccinated pigs, the amount of virus in the serum of pigs that become viremic is generally not much lower than the viral load in unvaccinated pigs (6, 8, 36). In consequence, we believe that the sensitivity of detection is unlikely to be responsible for the lack of virus detection in serum samples in our study. Another possibility is that the presence of an immune response at the time of challenge might have prevented free-virus viremia and only cell-associated viremia have occurred in those pigs. The existence of cell-associated viremia in the absence of free PRRSV in serum has been previously demonstrated in other studies in which a significant amount of circulating NAs was present (16) and could explain the arrival of the virus to lymphoid tissues. Unfortunately, buffy coat was not obtained in our study and, although we consider this a plausible and likely explanation, we cannot confirm it or discard it.

Finally, in the remaining three experimental groups in experiment A, only the classical partial protection was repeatedly reported in the literature upon detection of heterologous PRRSV exposure. In fact, these results can be considered the most likely outcome upon the exposure of previously infected pigs to a heterologous PRRSV isolate. Nonetheless, the differences with the results recorded for the other experimental groups in experiment A are outstanding. These differences might be explained, at least partially, by the lack of cross-reactive NAs at the time of challenge. However, it should be mentioned that the characteristics of the PRRSV isolates used for the secondary exposure in some of those groups might have also contributed to the outcome of infection. Thus, the pigs of group A.2 were exposed to a PRRSV-2 isolate, and cross-protection between PRRSV-1 and PRRSV-2 is considered insignificant, as demonstrated in different studies carried out in adult sows (38, 39), boars (40, 41), and piglets (42, 43). This is probably due to the enormous genetic divergence between the two PRRSV species (14). On the other hand, pigs of group A.3 were exposed to the heterologous PRRSV isolate EU-17, which is an Italian isolate with a relatively low genomic similarity with Sp-3a. Besides, this isolate has been classified as an isolate of high virulence based on the severity of the clinical signs and lesions recorded in a study carried out in 3-week-old pigs in the respiratory model of the disease (44). Virulence might be a relevant characteristic because it has been demonstrated that PRRSV-1 isolates of higher virulence replicate more efficiently in the host (45, 46) and in a wider range of cellular types (47, 48), and it can be speculated that this better *in vivo* replication might help to overcome any previously existing immunity, either humoral or cellular.

On the contrary, in experiment B, previous immunization of pigs with EU-11a had a limited or very limited effect on the replication of the heterologous PRRSV isolate. Thus, the percentage of positive serum samples in immunized pigs ranged from 22.9% to 85.7%, compared with frequencies of isolation from 87.7% to 100% in control pigs. This frequency of detection was, in general, higher than that reported in the literature when pigs immunized with different commercial vaccines have been later exposed to virulent challenges [e.g. 32% in vaccinated versus 84% in unvaccinated pigs in the study by Prieto et al. (8) or 10% to 12.5% in vaccinated *versus* 82.5% in unvaccinated pigs in the study by Kim et al. (42)]. Consistently, PRRSV was found in the tonsils of all pigs, regardless the group they belonged to.

The virus found in the tonsils of pigs in experiment B was the challenge virus, whereas in experiment A, not only the challenge virus but also the immunization virus was found, in this last case, in some non-viremic pigs. However, these results might be biased because of the method used for virus identification (i.e., ORF5 sequencing by Sanger method). PRRSV is known to persist in tonsils for prolonged periods of time (34), and different viruses can coexist in infected pigs for some time, even though predominant viruses tend to overcome all others and eventually make them disappear of the host (49). Consequently, it is possible that some pigs bore the immunization isolate at the time of challenge, and it became the minor population upon challenge. Besides, coexistence of different isolates might lead to the generation of mosaic viruses by recombination (50). Thus, immunization virus could have been present in tonsils in experiment B, but only the predominant challenge virus was detected by sequencing, and some of the detected viruses might have been recombinant viruses. Next-generation sequencing of full-genome would have helped to clarify these points but, unfortunately, it could not be carried out.

Taken together, the results of both experiments indicate that pigs immunized with Sp-3a controlled more efficiently a subsequent exposure to a heterologous PRRSV than pigs immunized with EU-11a. Although the ultimate reasons for these differences remain unknown, one remarkable difference between these two viruses is their ability to induce strain-specific and broadly reactive NAs. Thus, at the time of heterologous challenge (i.e., D70 of the experiment), the GMT of NAs against the homologous virus in pigs exposed to Sp-3a was 5.15 log_2_ compared with 2.06 log_2_ in the pigs exposed to EU-11a. However, it has to be bore in mind that one limitation of this measure is that SN assays have been performed in MARC-145 cell line, which is not a natural target cell for PRRSV. We selected MARC-145 cell line to carry out the SN assays for several reasons. First, all NA passive-transfer studies which have proven the role of NA in protection and have determined the minimum titer needed for protection in SN assays carried out in MARC-145 cell line (15, 16). In the same way, our previous study aimed to determine the existence of differences in the NA induction ability among PRRSV-1 isolates and used to select the PRRSV isolates to be used in the present study also used MARC-145 cell-line for SN assays (21). Thus, the use of this methodology allowed us to compare our results with previous studies. Besides, although there are some studies in the literature that have used PAM cells in SN assays (18, 51, 52), most of the PRRSV vaccination and challenge studies have used MARC-145 to carry out the SN assays used to determine the titer of NAs (53–55), and this is an accepted and well-established technique for the determination of NAs against PRRSV. Nonetheless, MARC-145 cells contain the simian CD163 instead of the porcine molecule, and we cannot rule out the possibility that the use of PAM or other modified porcine cells expressing porcine CD163 and sialoadhesin (Sn) or Siglec 10 (56) would have rendered slightly different results. Unfortunately, we could not do the comparison because of the logistical complications of testing a high number of samples in PAM and to the unavailability of CD163 and Sn transfected cells in our laboratory.

Nonetheless, we do believe that the differences found between isolates in their ability to induce both strain-specific and broadly reactive NAs, although could be qualified, are real, particularly considering the differences found in protection. We do not know the reasons why different viruses exhibit different NA-induction capability, but the ability to induce NAs seems to be an intrinsic property of PRRSV isolates as it has been described in the literature for PRRSV-1 (11, 21), as well as for PRRSV-2 (20, 57).

On the other hand, most of the NAs generated upon PRRSV infection seem to be strain-specific (20–22). However, to confer protection against heterologous reinfections, a PRRSV isolate must be able to induce cross-reactive NAs. When we looked at the cross-reactivity of NAs at the time of challenge, and consistently with our previous data, it was observed that the recognition of the heterologous viruses by NAs was better in experiment A than in experiment B. Thus, in experiment B, cross-reactive NAs were absent or barely detectable in most experimental groups. The only remarkable exception was group B.5, in which the GMT of the titer of NAs against the challenge virus was 1.64 log_2_ on D70 of the experiment. On the contrary, in experiment A, detectable titers of cross-reactive NAs were detected the day of challenge in six out of the eight experimental groups, with GMTs ranging from 0.72 to 2.93 log_2_.

The reasons why broadly reactive NAs are generated upon PRRSV infection remain undetermined. In our study, it could be speculated that the significantly lower level of homologous NAs developed in the pigs of experiment B compared with the pigs of experiment A (i.e. 2.06 log_2_ versus 5.15 log_2_) is the reason for the lack of cross-reactivity observed in experiment B. However, in our opinion, the low level of homologous NAs does not fully explain the lack of cross-reactivity of NAs specific for EU-11a. We base this statement in two facts. First, in a previous study in which the homologous titer of a hyperimmune serum against EU-11a was fixed at 7 log_2_, heterologous isolates were barely recognized in SN assays (21). Second, in the present study, the sera of the few pigs that had homologous titers of NAs between 4 and 6 log_2_ the day of challenge in experiment B reacted poorly with the challenge strain (data not shown). Both results indicate that most NAs generated upon primoinfection with EU-11a are strain-specific.

A number of NEs, some of them linear and other conformational, have been described in different PRRSV structural proteins, including GP2, GP3, and GP4 (52) of PRRSV-1, GP5 of PRRSV-1 and PRRSV-2 (58, 59), and M protein of PRRSV-2 (60). However, their role in the induction of broadly reactive NAs is largely unknown (10). Only the NE identified in the M protein of PRRSV-2 has been proven to play a role in broadly neutralizing activity, with a single amino acid necessary for the broad recognition (22). However, as the role of M protein in the induction of NAs in PRRSV-1 remained largely unidentified, it is not possible to know how important this NE is in the induction of broadly reactive NAs in the case of PRRSV-1. On the contrary, an NE located in the ectodomain of GP4 is considered immunodominant and responsible for the induction of most of the NAs elicited upon infection (61). However, this NE is highly variable (61), and most isolates display different sequences and even deletions in this epitope (21), probably because of a selective pressure during *in vivo* replication (51). The preferential pig response against this highly variable NE could help to explain the poor recognition of heterologous viruses upon PRRSV infection (21) and, although the specificity of the NAs generated against EU-11a was not identified, it could be speculated that the NA response against this virus was mainly directly against this epitope. On the contrary, it is likely that Sp-3a exposes more efficiently unidentified conserved NE, relevant for the recognition of heterologous viruses. Unfortunately, up-to-date conserved NE have not been mapped, with the only exception of the already mentioned NE in M protein of PRRSV-2, and this lack of knowledge makes it very difficult to identify the particular sequence or sequences recognized by the pigs exposed to Sp-3a virus.

Regardless the reasons that explain the differences in the generation of broadly reactive NAs between the two viruses used in our experiments, protection was better in groups in which NAs against the heterologous virus were detected on the day of the challenge. Thus, in experiment B, the initial immunization with EU-11a elicited very low levels of cross-reactive NAs and conferred very limited protection against a challenge with heterologous PRRSV. However, viremia was less frequently found in group B.5, in which cross-reactive NAs were detected (although at low titers, i.e. GMT of NAs = 1.64 log_2_) than in any other group.

On the contrary, in experiment A, an outstanding level of protection, with a complete prevention of viremia, was observed in five of the eight experimental groups. Consistent with this finding, relatively significant titers of cross-reactive NAs were detected the day of challenge in those five groups, with GMTs of NAs ranging from 1.78 to 2.93 log_2_.

Nonetheless, the levels of cross-reactive NAs found in our study are below the cutoff described in the literature for protection against viremia (i.e., 1:8 or 3 log_2_). There might be several explanations for this finding. First of all, it is possible that NAs titers close to 3 log_2_, which has been proven to be sufficient to protect sows from infection (15), are closer to the amount needed to confer complete protection, or at least to prevent viremia, in 140-day-old pigs than in young piglets. These differences can be explained by a higher maturity of the immune system and, most likely, by the lower susceptibility to infection of the main target cell of PRRSV, PAM (62, 63). Thus, it is conceivable that the lower viral load typically found in adults (64) requires lower titers of NAs for an efficient control of the infection (16).

On the other hand, in this study, cross-reactive NAs were generated by natural infection instead of being passively transferred. That means that it is possible that NAs were also present in the mucosal surfaces, blocking the virus at the portal of entry and avoiding the establishment of infection or, at least, viremia, regardless the titer of NAs in bloodstream. In this line of thinking, it has been demonstrated that upon infection, local immunity develops and PRRSV-specific antibodies can be found in respiratory secretions (65), oral fluids, or bronchoalveolar lavages (66).

Finally, it is likely that other components of the immune response, and particularly, PRRSV-specific cellular immune response, have developed upon immunization and contributed to confer protection against a secondary heterologous PRRSV exposure. This theory could also explain the high level of protection observed in a few pigs in which cross-reactive NAs titers were lower than 2 log_2_ (data not shown). This observation has been made in other studies, and protection has been attributed to cell-mediated immunity (11, 12). Unfortunately, because of economic, logistic, and labor constrictions, it was not possible to study the role of the cell-mediated immune response in the protection observed, but we consider that cell-mediated immunity might also have contributed significantly to the control of the heterologous viruses.

Nonetheless, and regardless the contribution of cell-mediated immunity to protection, it is remarkable that in two of the three groups of experiment A in which only partial protection was achieved, based on the recording of viremia in some pigs, cross-reactive NAs were not detected at the time of challenge, whereas in the third group (i.e., group A.4), only very low titers of NAs (i.e. GMT of NAs = 0.72 log_2_) were found. Even more, in the only pig of this group with cross-reactive NAs of 2 log_2_ at the time of challenge, viremia was never detected, and the tonsil was negative for PRRSV (data not shown), supporting the notion that NAs played a relevant role in protection in our study.

Another interesting finding is the evolution of the NA titer against the virus used for immunization (i.e., homologous) and for challenge (i.e., heterologous) in the different experimental groups. Thus, in experiment A, a booster against homologous virus upon challenge was detected in the three groups in which viremia was detected (although differences in NA titers were statistically significant only in groups exposed to EU-17 and Sp-27) and NAs titers remained unchanged in all other groups. On the contrary, in experiment B, a booster was recorded in all experimental groups, and the differences in NA titers were statistically significant in all groups with the only exception of pigs exposed to Sp-22. These results indicate that the viremia subsequent to reinfection induces a secondary immune response, whereas the absence of viremia, either due to sterilizing immunity or accompanied by the distribution of the virus to lymphoid tissues, does not change significantly the titer of NAs. Thus, the presence or absence of a secondary immune response might be an indirect indicator of protection.

Finally, it is unknown why some PRRSV-1 isolates generate cross-reactive NAs, whereas others do not. The results of this study encourage further studies aimed to characterize the intrinsic properties of PRRSV-1 isolates that stimulate the generation of broadly reactive NAs. In the same way, the two PRRSV isolates characterized in this study represent a very useful tool to decipher the components of the immune response essential for the development of a protective immunity against PRRSV. Finally, the results of this study and the role of broadly reactive NAs in protection should be confirmed in further studies using a significant number of heterologous PRRSV isolates with different biological properties and measuring cell-mediated immunity to establish its role in protection.

## Summary/Conclusion

In conclusion, the results reported here confirm that PRRSV-1 isolates differ in their ability to induce cross-reactive NAs and in their ability to confer protection against heterologous reinfections. According to the results of our study, PRRSV-1 isolates able to induce broadly reactive NAs as determined *in vitro* could confer a better level of virological protection against heterologous PRRSV infection *in vivo*. However, it has to be mentioned that the correlation between the level of NAs and protection was not perfect and that a few pigs without measurable titers of NAs or with NA titers lower than those established as the threshold for protection in passive transfer studies were protected. This remarkable level of protection could be explained by the contribution of other components of the immune response, as cell-mediated immunity or mucosal immunity. Nonetheless, the results of this study seem to confirm that NAs, and particularly broadly reactive NAs, could play a role in protection against PRRSV reinfections.

## Data Availability Statement

The raw data supporting the conclusions of this article will be made available by the authors, without undue reservation.

## Ethics Statement

The animal study was reviewed and approved by Comité de Experimentación Animal. Universidad Complutense de Madrid. Madrid, España.

## Author Contributions

JC and CP conceived, designed and coordinated the study. FM-L, FD-F, IS, and CP carried out the experiments. FM-L, JC, and CP analyzed and interpreted the experimental results. FM-L wrote the draft of the manuscript. FM-L, JC, and CP reviewed the original draft of the manuscript. All authors contributed to the article and approved the submitted version.

## Funding

This study was supported by grants AGL2007-66695, AGL2008-05708-C02 and CSD-2006-00007 from the Spanish Government and FEI 20/39 from the University Complutense of Madrid. FM-L and F-DF were funded by Project Consolider-Ingenio 2010 CDS2006-00007.

## Conflict of Interest

The authors declare that the research was conducted in the absence of any commercial or financial relationships that could be construed as a potential conflict of interest.

## Publisher’s Note

All claims expressed in this article are solely those of the authors and do not necessarily represent those of their affiliated organizations, or those of the publisher, the editors and the reviewers. Any product that may be evaluated in this article, or claim that may be made by its manufacturer, is not guaranteed or endorsed by the publisher.
